# Birth defects data from hospital-based birth defect surveillance in Guilin, China, 2018–2020

**DOI:** 10.3389/fpubh.2022.961613

**Published:** 2022-08-24

**Authors:** Xingdi Yang, Jianjuan Zeng, Yiping Gu, Yiming Fang, Caiyun Wei, Shengkui Tan, Xiaoying Zhang

**Affiliations:** ^1^Public Health, Guilin Medical University, Guilin, China; ^2^The Guangxi Key Laboratory of Environmental Exposomics and Entire Lifecycle Heath, Guilin, China; ^3^Guangxi Health Commission Key Laboratory of Entire Lifecycle Health and Care, Guilin, China; ^4^Department of Child Health Care, Guilin Maternal and Child Health Hospital, Guilin, China

**Keywords:** birth defects, hospital-based surveillance, epidemiology, etiology, congenital heart defects, neural tube defects

## Abstract

**Objectives:**

Birth defects (BDs) are a major contributor to perinatal and infant mortality, morbidity and lifelong disability worldwide. A hospital-based study on birth defects was designed in Guilin city in the Guangxi province of Southwestern China aiming to determine the prevalence of BDs in the studied region, and the classify the BDs based on clinical presentation and causation.

**Methods:**

The study involved BDs among all pregnancy outcomes (live births, stillbirths, death within 7 days, and pregnancy terminations) born in the 42 registered hospitals of Guilin between 2018 and 2020. The epidemiological characteristics of BDs and the etiologic profile of BDs were evaluated in this study.

**Results:**

Of the total 147,817 births recorded during the study period, 2,003 infants with BDs were detected, giving a total prevalence rate of 13.55 per 1,000 births. The top five BD types were congenital heart defects, polydactyly, syndactyly, malformations of the external ear, and talipes equinovarus, whereas, neural tube defects, congential esophageal atresia, gastroschisis, extrophy of urinary bladder, were the least common BD types in these 3 years. Only 8.84% of cases were assigned a known etiology, while most cases (91.16%) could not be conclusively assigned a specific cause.

**Conclusion:**

This study provides an epidemiological description of BDs in Guilin, which may be helpful for understanding the overall situation in Southwest China of BDs and aid in more comprehensive studies of BDs in future healthcare systems, including funding investment, policy-making, monitor, prevention. Strong prevention strategies should be the priority to reduce BDs and improve the birth quality.

## Introduction

Birth defects (BDs) are defined as structural, functional, and/or biochemical-molecular anomalies that occur during the development of the embryo or fetus (1–3). BDs remain a major contributor to perinatal and infant mortality, morbidity and lifelong disability worldwide, and represent a major public health problem because they cause substantial personal, social, and financial burden, in addition to having a considerable impact on the population quality and social development (4, 5).

Hospital-based and population-based surveillance are the two main types of national BD surveillance systems (4, 6). In China, most BD research data are obtained from hospital-based systems (6, 7). According to the requirements of the Ministry of Health of China, the “Maternal and Child Health Monitoring Manual in China” monitors BDs among all early fetuses <28 weeks of gestation and perinatal infants between 28 weeks of gestation and 7 days after birth who are born in hospitals, and also collects maternal information, such as age, gravidity, parity, and physical condition (1, 3).

The overall prevalence of BDs might have increased, and approximately 3–5% of births worldwide are affected by BDs (2, 4). In China, the prevalence of BD ranges from 0.715 to 19.184% (8). Recently, increase attention has been paid to BDs in low-income provinces of southwestern China. Guilin is an underdeveloped city in the low-income province of Guangxi in southwestern China. In 2019, its cumulative gross domestic product was approximately CN ¥ 210.556 billion, which was much lower than the national per capita value in the same period, ranking 132 in mainland China, and becoming one of the key BD surveillance spots.

Furthermore, commonly used BD classification schemes were International Statistical Classification of Diseases and Related Health Problems (ICD), such as 9th or 10th revision (ICD-9, or ICD-10) (2, 9, 10). ICD is the standard used globally to classify diseases including birth defects. They based on clinical presentation, typically organized by anatomy or function, they are valuable for general purposes such as studies on morbidity and mortality, but not ideal in the evaluation of etiologies (2). Thus, we conducted a study to describe the epidemiological characteristics of BDs in Guilin between 2018 and 2020, along with classification of BDs based on clinical presentation and etiologic profile of BDs.

## Materials and methods

### Data collection

The data source for this study was the “Maternal and Child Health Monitoring Manual in China” from Guilin between 2018 and 2020; 42 hospitals were registered in the system. In this retrospective study, we only used the monitoring data without contacting or identifying the patients.

BDs were diagnosed by physical examination, ultrasonography, X-ray examination, and/or genetic diagnostic methods, based on the Chinese National Criteria of Birth Defects and Tiny Deformities and the clinical modification codes as congenital malformations, deformations, and chromosomal abnormalities (codes Q00–Q99) of ICD-10 (1, 6, 10). Experts from each registered hospital were responsible for the diagnostic confirmation and provision of technical support. Trained gynecological and pediatric or neonatal doctors were responsible for filling the case card of each infant with BD in the “Birth Defects Registration Form,” and uploading it into the surveillance system both on paper and online. In addition, each case card recorded maternal information (including her age, gravidity, parity, education, economic status, physical condition, and medication use during pregnancy), and we used this information to analyze the potential etiological profile of BDs in this study.

The study involved BDs among all pregnancy outcomes (live births, stillbirths, death within 7 days, and pregnancy terminations) during perinatal period (between 28 weeks of gestation and seven days after birth) born in the 42 registered hospitals of Guilin. Live births included singleton, twin and multiple live births. Stillbirth was defined as fetal death at 28 weeks estimated gestational age or more. Death within 7 days included neonatal death within the first 7 days after birth. If pregnant women with a prenatal diagnosis of a defective fetus wanted to terminate their pregnancies, they had to go to medical institutions that were qualified for prenatal diagnosis, and their medical records of BDs had to be ascertained and filled in the registration forms for BDs.

The studies were reviewed and approved by the Medical Ethics Committee of Guilin Medical University (number: GYLL2021012).

### Classification of BDs based on clinical presentation

In this study, we first classified BDs based on the ICD-10 system to better understand the epidemiological characteristics of BDs (1, 10). Thus, nine categories were involved in the classification as the research outcome. The nine categories were the nervous system, the eyes, ears, face, and neck, the digestive system, the genital and urinary organs system, the musculoskeletal system, the circulatory system, the respiratory system, genetic abnormality, and other BD types. Infants with BDs from more than one defect category were included in each applicable major defect category (4).

### Etiologic classification

In this study, we further implemented an etiological (known, unknown) classification to systematically capture the etiology of BDs. Briefly, the known etiology included four main criteria: genetic, environmental, gene-environment interactions and twinning (2, 11–15). The genetic etiology included chromosomal abnormalities (number and structure), single gene abnormalities and family history of malformations (2, 13, 16, 17). Environmental etiology included various types of environmental exposure recognized as human teratogens, such as, maternal infections (such as viruses, bacteria, and protozoa) (2, 12, 18), maternal medications (such as antibiotics and valproic acid) (2), occupational exposure (such as X-ray, agricultural pesticides, chemicals) (12), maternal illness (such as diabetes) (2, 19), and maternal smoking or alcohol (12, 13), or multiple known environmental factors exposures. Gene-environment interactions etiology included complex genetic and environmental interactions such as mother had family history of malformations as well as environmental exposure (14, 15). The twinning etiology included acardiac and conjoined twins (2).

### Statistical analysis

Statistical analyses were performed using SPSS version 22.0. The prevalence rates of BDs were calculated as the total number of infants with BD cases (live births, stillbirths, death within 7 days, pregnancy terminations) divided by the total number of births (live births and stillbirths). The prevalence rates of BDs were calculated both across all years and for each year separately. The prevalence rates of different types of BDs were calculated and ranked in a descending order, separately. Infants with BDs from more than one defect category were included in each applicable major defect category for classification and ranking (4). The chi-square test was used to test the between-group differences. The etiological classification of birth defects was shown as case counts in each category. *P* < 0.05 was considered statistically significant.

## Results

### Annual prevalence rates of BDs in Guilin from 2018 to 2020

This hospital-based study included 2,003 infants with BDs from among a total of 147,817 infants, giving a total prevalence rate of 13.55 per 1,000 births. The prevalence rate of BDs increased from 12.61 per 1,000 births (706/56,006) in 2018 to 15.53 per 1,000 births (648/41,740) in 2020 (overall: χ^2^ = 17.21, *P* = 0.000; 2018 vs. 2019: χ^2^ = 0.27, *P* = 0.61; 2019 vs. 2020: χ^2^ = 10.74, *P* = 0.001; 2018 vs. 2020: χ^2^ = 14.92, *P* = 0.000; Table 1). In this study, two time periods for diagnosis, i.e., “prenatal diagnosis” and “postpartum diagnosis within 7 days” were defined for BD screenings, and most BDs were diagnosed in live births in the time period of “postpartum diagnosis within 7 days.” Thus, “postpartum diagnosis within 7 days” was the main diagnosis time period for screening BDs in Guilin (Table 1).

**Table 1:** Birth defects counts and prevalence (per 1,000 births) stratified by pregnancy outcome at different two diagnosis periods in Guilin, 2018–2020.

**Diagnosis period**	**Pregnancy outcome**	**2018**		**2019**		**2020**		**Total**	
		**(** ***N*** **: 56,006 births)**		**(** ***N*** **: 50,071 births)**		**(** ***N*** **: 41,740 births)**		**(** ***N*** **: 147,817 births)**	
		***n***	**Prevalence**	***n***	**Prevalence**	***n***	**Prevalence**	***n***	**Prevalence**
Prenatal diagnosis	Live births	34	0.61	39	0.78	44	1.06	117	0.79
	Stillbirths	1	0.02	0	0.00	0	0.00	1	0.01
	Death within 7 days	0	0.00	0	0.00	0	0.00	0	0.00
	Pregnancy terminations	68	1.21	40	0.80	37	0.89	145	0.98
	Total	103	1.84	79	1.58	81	1.95	263	1.78
Postpartum diagnosis within 7 days	Live births	596	10.64	566	11.30	559	13.48	1,721	11.64
	Stillbirths	0	0.00	0	0.00	0	0.00	0	0.00
	Death within 7 days	3	0.05	3	0.06	4	0.10	10	0.07
	Pregnancy terminations	4	0.07	1	0.02	4	0.10	9	0.06
	Total	603	10.77	570	11.38	567	13.58	1,740	11.77
Total		706	12.61	649	12.96	648	15.53	2,003	13.55^*^

N: number; n: number;

* P < 0.05, Pearson chi-square test was used, χ^2^ = 17.21, P = 0.000, there were significant differences among the three groups (2018, 2019 and 2020), furthermore, there was no significant difference between 2018 and 2019 groups (P = 0.61), but there was significant difference between 2019 and 2020 groups (P = 0.001), or between 2018 and 2020 groups (P = 0.000).

### Types of BDs based on clinical presentation

We found that the most common BDs involved the circulatory, musculoskeletal, facial, urogenital, genetic and digestive systems (Table 2). In the 3 years from 2018 to 2020, the five most common BDs were congenital heart defects (CHD), polydactyly, syndactyly, malformations of the external ear, and talipes equinovarus, with total prevalence rates of 3.43, 3.22, 1.14, 1.08, and 0.91 per 1,000 births, respectively. The total prevalence rate of CHD was the highest among all BDs, and its annual prevalence rate increased from 2.61 per 1000 births in 2018 to 4.56 per 1000 births in 2020 significantly, which is a significant increase (overall: χ^2^ = 26.48, *P* = 0.000; 2018 vs. 2019: χ^2^ = 5.80, *P* = 0.016; 2019 vs. 2020: χ^2^ =7.51, *P* = 0.006; 2018 vs. 2020: χ^2^ = 26.41, *P* = 0.000; Table 2). Among the CHD cases, the top five common subtypes of CHD in Guilin (2018–2020) were atrial septal defect, patent ductus arteriosus, ventricular septal defect, atrioventricular septal defect and Tetralogy of Fallot (Supplementary Table 1). However, neural tube defects (NTD), congential esophageal atresia, gastroschisis, extrophy of urinary bladder, were the least common BDs in these 3 years (total prevalence: 0.04, 0.03, 0.02, and 0.01 per 1,000 births, respectively). The overall prevalence rates and ranks of all observed BDs are presented in Table 2.

**Table 2:** Prevalence rates of different types of birth defects in Guilin, 2018–2020 (per 1,000 birth).

**Types of BDs**	**2018**		**2019**		**2020**		**Total**	
	**(*****N:*** **56,006 births)**		**(*****N:*** **50,071 births)**		**(*****N:*** **41,740 births)**		**(*****N:*** **147,817 births)**	
	**Prevalence**	**Rank**	**Prevalence**	**Rank**	**Prevalence**	**Rank**	**Prevalence**	**Rank**
**Nervous system (Q00–Q07)**								
Neural tube defect^ǀ^	0.10	17	0	–	0.02	16	0.04	21
Congenital hydrocephalus	0.14	16	0.14	15	0.17	13	0.15	16
Others^†^	0.18	13	0.18	14	0.17	13	0.18	15
**eyes, ear, face and neck (Q10–Q18)**								
Small ears (or no ears)	0.16	14	0.18	14	0.22	11	0.18	15
Other malformations of external ear	1.25	3	0.82	4	1.03	5	1.08	4
Others^‡^	0.09	18	0.10	16	0	–	0.07	18
**Digestive system (Q35** ***–*** **Q37, Q38** ***–*** **Q45)**								
Cleft palate	0.21	12	0.34	10	0.38	8	0.30	9
Cleft lip	0.21	12	0.30	11	0.19	12	0.24	12
Cleft lip and palate	0.27	9	0.38	9	0.19	12	0.28	10
Congential esophageal atresia	0.04	21	0.02	19	0.02	16	0.03	22
Congenital atresia of rectum and anus	0.25	10	0.28	12	0.07	14	0.21	13
Others^§^	0.05	20	0.10	16	0.05	15	0.07	18
**Genital and urinary organ systems (Q50** ***–*** **Q56, Q60** ***–*** **Q64)**								
Hypospadias	0.46	7	0.62	6	0.72	7	0.59	7
Extrophy of urinary bladder	0	–	0.02	19	0.02	16	0.01	24
Congenital malformation of kidney	0.16	15	0.20	13	0.19	12	0.18	15
Others^¶^	0.32	8	0.18	14	0.24	10	0.25	11
**Musculoskeletal system (Q65–Q79)**								
Talipes equinovarus	0.93	5	0.74	5	1.10	4	0.91	5
Polydactyly	3.09	1	3.02	2	3.71	2	3.24	2
Syndactyly	1.00	4	1.32	3	1.13	3	1.14	3
Short limbs	0.21	12	0.18	14	0.19	12	0.20	14
Congenital diaphragmatic hernia	0.07	19	0.02	19	0.05	15	0.05	20
Omphalocele	0.02	22	0.02	19	0.17	13	0.06^*^	19
Gastroschisis	0	–	0.02	19	0.05	15	0.02	23
Others^ǁ^	0.66	6	0.52	7	0.91	6	0.68	6
**Circulatory system (Q20** ***–*** **Q28)**								
Congenital heart defects^∵^	2.61	2	3.42	1	4.56	1	3.43^*^	1
**Genetic abnormality (Q90** ***–*** **Q99)**								
Down's syndrome (Trisomy 21 syndrome)	0.23	11	0.02	19	0.02	16	0.10^*^	17
Others^$^	0.32	8	0.40	8	0.38	8	0.37	8
**Respiratory system (Q30** ***–*** **Q34)**								
Congenital malformation of larynx, trachea and lung	0.04	21	0.08	17	0.02	16	0.05	20
**Other BDs (Q80** ***–*** **Q89)**								
Hemangioma	0.05	20	0.04	18	0.07	14	0.05	20
Others	0.46	7	0.3	11	0.34	9	0.37	8

ǀ anencephaly, spina bifida and encephalocele.

† Agenesis of the Corpus Callosum, Dural sinus malformation, Intracranial abnormality, cerebellar malformation, microcephalia, encephalodysplasia, etc.

‡ Nasal bone absence, Congenital torticollis, Congenital absence of eyeball in both eyes, Congenital mandibular deformity, etc.

§ Congenital esophago tracheal fistula, Congenital intestinal dilatation, Rectum to skin fistula, Congenital gastric volvulus, Congenital atresia of intestine, Congenital pyloristenosis, Gastrointestinal obstruction, etc.

¶ Congenital anomalies of the kidney and urinary tract, Congenital abnormalities of external genitalia (male/female), Cryptorchidism, Small penis, etc.

ǁ strephexopodia, Hallux valgus, Congenital dislocation of the knee, Pectus Excavatum, Arthrogryposis multiplex congenital, Bone dysplasia, etc.

∵ atrial septal defect, patent ductus arteriosus, Tetralogy of Fallot, endocardium cushion defect, transposition of the great vessels, pulmonary valve atresia and stenosis, complex congenital heart disease, hypoplastic left heart syndrome, coarctation of the aorta, tricuspid valve atresia and stenosis, aortic valve stenosis, total anomalous pulmonary venous connection, single ventricle interrupted aortic arch, and double outlet right ventricle, single ventricle, primary pulmonary hypertension, etc.

$ 47,XXX/XXY, Trisomy 18, Trisomy 13, Turner Syndrome, Chromosome 3 abnormality, Chromosome 5 abnormality, Chromosome 11 abnormality, Chromosome 17 abnormality, Other chromosomal mosaic, Other chromosome deletions / duplications, DiGeorgr Syndrome, Single gene abnormalities (such asα- or β-thalassemias genes, ATP1A3,), etc.

* P < 0.05.

Omphalocele: Fisher chi-square test was used, χ^2^ = 8.50, P = 0.007, there were significant difference among the three groups (2018, 2019 and 2020), furthermore, there was no significant difference between 2018 and 2019 groups (P = 1.00), but there was significant difference between 2018 and 2020 groups (P = 0.025) or between 2019 and 2020 groups (P = 0.027).

Congenital heart defects: Pearson chi-square test was used, χ^2^ = 26.48, P = 0.000, there were significant difference among the three groups (2018, 2019 and 2020), furthermore, there was significant difference between each two groups (2018 and 2019 groups: P = 0.016, 2019 and 2020 groups: P = 0.006, or 2018 and 2020 groups: P = 0.000).

Down's syndrome: Fisher chi-square test was used, χ^2^ = 13.46, P = 0.000, there were significant difference among the three groups (2018, 2019 and 2020), furthermore, there was no significant difference between 2019 and 2020 groups (P = 1.00), but there was significant difference between 2018 and 2019 groups (P = 0.002) or between 2018 and 2020 groups (P = 0.006).

### Etiologic classification of BDs

Overall, 8.84% of cases were assigned a known etiology; among the known etiology group, 177 cases were further classified into genetic etiology (97/177), environmental etiology (74/177), and gene-environmental etiology (6/177) (Table 3). As shown in Table 3, cases with a known etiology were mostly associated with chromosomal abnormalities (*n* = 54, 30.51%), family history (*n* = 34, 19.21%), maternal diabetes (*n* = 30, 16.95%), and maternal infection (*n* = 22, 12.43%). However, most cases (91.16%) could not be conclusively assigned a specific cause, which underscored the current knowledge gaps and challenges in BD prevention.

**Table 3:** Etiologic classification of birth defects in Guilin, 2018–2020.

	**2018**	**2019**	**2020**	**Total**
**Known etiology**				
*N* (%) total	92 (13.03%)	50 (7.70%)	35 (5.40%)	177 (8.84%)
**Genetic**	48	28	21	97
Chromosomal abnormality^ǀ^	27	16	11	54
Single gene abnormality	2	3	4	9
Family history^†^	19	9	6	34
**Environmental**	39	21	14	74
Maternal infecton^‡^	12	6	4	22
Maternal medication^§^	2	4	2	8
Occupational exposure^#^	4	1	-	5
Diabetes	18	6	6	30
Maternal smoking	-	1	-	1
Maternal alcohol	-	-	-	-
Multiple environmental interactions	3	3	2	8
**Gene- Environment interactions**	5	1	-	6
**Twinning**	-	-	-	-
Acardiac	-	-	-	-
Conjoined	-	-	-	-
**Unknown etiology**				
N (%) total	614 (86.97%)	599 (92.30%)	613 (94.60%)	1,826 (91.16%)

N, number.

ǀ Including number (trisomy, mosaic) or structure (insertion, deletion) abnormalities.

† Including history of malformations within the family or siblings.

‡ Including virus (herpesvirus, common cold, varicella, rubella, Epstein-Barr virus, cytomegalovirus, and influenza), bacteria infection (mycoplasma pneumoniae, Treponema pallidum pallidum), or protozoa (Toxoplasma gondii) infection.

§ Including antibiotics, sulfonamides, psychiatric medication (antiepileptic drugs, valproic acid), analgesic/antipyretic drug (acetaminophen), digoxin, or dexamethasone, excluding cases of diabetes mellitus.

# Including agricultural pesticides, X-ray, or chemical emission.

-: not detected.

## Discussion

In this hospital-based study, we used Guilin City's perinatal BD monitoring data to describe the epidemiological characteristics of common BDs and review the etiological identification of these BDs in Guilin, Guangxi province, China. The total prevalence of BDs was 13.55 per 1,000 births from 2018 to 2020, which was lower than the prevalence rate of 19.53 per 1,000 births in Liuzhou, another city in Guangxi (20).

Among the BDs, we found that CHD was the most common type of BD and its prevalence rate increased from 2.61 per 1000 births in 2018 to 4.56 per 1,000 births in 2020, the increase trend was consistent with the results of most previous studies in China and other countries (1, 9, 21). This phenomenon can be explained to some extent by the increasing proportion of older pregnant women, increasing social or natural environmental pressure, improvement in diagnostic techniques, for example, B-ultrasound, and advances in the ascertainment of CHD (1, 9). Although CHD was the most common type of BD in Guilin, the prevalence of CHD (total: 3.43 per 1,000 births) was still much lower than the global rate of 8–10 per 1,000 births (9). The low prevalence of CHD in Guilin may be due to several reasons. First, specialized BD monitoring and diagnosis centers in Guilin hospitals are underserved, with a scarcity of professional B-ultrasound physicians and specialist clinics, which can also be observed in other underdeveloped cities or countries. Second, in our study, data were collected from 28 weeks of gestation to 7 days after birth; however, some CHD cases may not be discovered during this period. Herein, CHD cases were mostly detected in the “Postpartum diagnosis within 7 days” (89.15%) (Supplementary Table 2). In the USA, the follow-up time for CHD cases is up to at least 1 year or without age limitation (4, 9). Third, some low-income pregnant women delivered in the hospital without any prenatal or postpartum diagnosis of CHD, resulting in missing or incomplete information. Our finding indicate that government should take action to reduce the prevalence of CHD: prioritize the prevention investment of BDs in healthcare system, promote CHD diagnosis and monitoring techniques innovations, strengthen training of related physicians in underdeveloped cities, narrow gaps of techniques between developed and underdeveloped cities/regions, increasing women's awareness of CHD.

Anencephaly, spina bifida and encephalocele, which are the three main types of neural tube defects (NTDs) (5, 22, 23). Mandatory folic acid supplementation is highly likely to be responsible for the NTDs being one of the least common BDs, which had also been observed in our study and other reports (3, 22). Forci K et al. (22) reported that a prevalence rate of NTD in Moroccan (2011–2016) was 1.00 per 1,000 births, which was a lower rate than those reported by previous Moroccan studies (e.g. 2008–2011: 1.21–2.18 per 1,000 births), and the decrease in NTDs in Morocco was due to the folic acid supplementation since 2008. Xie D et al. (3) reported that the prevalence rate of NTD in Hunan province of China declined from 1.44 per 1,000 births in 2005 to 0.33 per 1,000 births in 2014 since the Chinese government prompted folic acid supplement (3). In this study, we also found that the total prevalence rate of NTD in Guilin city of China was 0.04 per 1,000 births (2019–2020), this low prevalence rate was also due to the mandatory folic acid supplementation in Guilin since 2009. For example, in our study, about 99.00% of pregnant women in the 42 registered hospitals were supplemented with folic acid in 2019 (data not shown). NTD cases were always detected by prenatal diagnostic tests and followed by therapeutic termination of pregnancy (3, 22). Five of the six NTD cases (83.55%) in our study were diagnosed antenatally; all five cases (100%) underwent a pregnancy termination procedure (Supplementary Table 3). Furthermore, NTD cases have been reported to be associated with other malformations, and the rate of NTD-associated malformations varies from 12% to over 80% (5, 22, 24). In our study, half of the NTD cases (three cases) had multiple malformations, one anencephaly associated with cleft lip and palate, one spina bifida associated with congenital atresia of the rectum and anus, and one encephalocele with craniotabes (Supplementary Table 3), which demonstrate the importance of improving the prenatal diagnosis to reduce fetal BDs. Together, this study suggest that the prevention and control strategies implemented by the Ministry of Health should be further strengthen by enforcing folic acid supplementation, increasing women's awareness of BDs, providing more adequate medical care for pregnancy women, such as prenatal diagnosis (22).

Another highlight of this study was that we classified the common BDs according to their etiology. Determination of etiology is crucial for focusing research efforts on understanding the current gaps in knowledge, controlling the risk factors, and strengthening preventive measures. Few studies have attempted to estimate the proportion of BDs with or without a known etiology. In this study, we considered the etiology as known only if there was conclusive evidence that the etiology was associated with BD. However, some well-known risk factors, such as epidemiologic indicators (older age, multiple pregnancies, etc.), may be applicable to populations, but not to individual cases; therefore, these risk factors were not considered as known etiological factors (2). Twinning is another important criterion of known etiology (2), but we had no twinning sample; therefore, in this study, identifiable etiology consisted of genetic etiology, environmental etiology, and gene-environment interactions etiology. More and more attention has been paid to the role of gene-environment interactions as one of the causes for BDs (14, 15). In our study, six cases (6/2003) were found to be exposed to gene-environment interactions. Unfortunately, only 177 cases (8.84%) were assigned a known etiology in Guilin in 2018–2020, and the low proportion of BDs with known etiology may be attributed to several reasons. The first reason may be the low level of economic and technological development in Guilin; for example, although an increasing in the prevalence of genetic abnormalities in CHD patients was reported recently (2, 9), only 1 in 507 CHD patients underwent genetic testing in Guilin (data not shown). Second, there was incomplete data collection and specialists in registered hospitals did not record some exposures (such as maternal infection, medication, pregestational diabetes) or query relevant information. Third, most BDs are caused by complex gene-environment interactions, and the causes of these BDs are still unknown (11). There are large gaps in the current knowledge on the causes of BDs, which underscores the importance of accelerating basic and translational research.

## Conclusion

In conclusion, this study provides an epidemiological description of BDs in Guilin: a total prevalence rate of BDs was 13.55 per 1,000 births, CHD was the top common type of BD (3.43 per 1,000 births), while NTD was well controlled, and the prevalence rate was only 0.04 per 1,000 births; 177 cases were assigned a known etiology, including genetic etiology, environmental etiology, and gene-environmental etiology. These data may be helpful for understanding the overall situation of BDs in Southwest China and aid in more comprehensive studies of BDs in future healthcare systems, including funding investment, policy-making, monitor, prevention. In order to reduce BDs, improve the birth quality, and promote developmental health, government should prioritize the prevention investment of BDs in healthcare system, promote diagnosis and monitoring techniques innovations, narrow gaps of techniques between developed cities/regions and underdeveloped cities/regions. And the prevention strategy implemented by the Ministry of Health should be further strengthen by enforcing essential nutrients (such as folic acid) supplementation, increasing women's awareness of BDs, providing more adequate medical care for pregnancy women, such as prenatal diagnosis.

## Data availability statement

The raw data supporting the conclusions of this article will be made available by the authors, without undue reservation.

## Ethics statement

The studies involving human participants were reviewed and approved by Medical Ethics Committee of Guilin Medical University. Written informed consent from the participants' legal guardian/next of kin was not required to participate in this study in accordance with the national legislation and the institutional requirements.

## Author contributions

ST and XZ contributed to data collection, analysis, interpretation, and manuscript preparation, full access to all of the data in the study and serves as the guarantor of the manuscript. XY, JZ, YG, YF, and CW collected the data, analyzed the data, and manuscript preparation. XZ and CW obtained fundings. All authors contributed to the study conception, design, and read and approved the final manuscript.

## Funding

This study was funded by the National Natural Science Foundation of China (82160620), the Science and Technology Base and Talent Project of Guangxi Province (AD19245006), and the National College Students Innovation and Entrepreneurship Training Program (202110601027).

## Conflict of interest

The authors declare that the research was conducted in the absence of any commercial or financial relationships that could be construed as a potential conflict of interest.

## Publisher's note

All claims expressed in this article are solely those of the authors and do not necessarily represent those of their affiliated organizations, or those of the publisher, the editors and the reviewers. Any product that may be evaluated in this article, or claim that may be made by its manufacturer, is not guaranteed or endorsed by the publisher.

## Abbreviations

BD: Birth defect
CHD: congenital heart defect
NTD: neural tube defect.
