# Supplementary material for: Birth defects data from hospital-based birth defect surveillance in Guilin, China, 2018–2020
Source: Front Public Health. 2022 Aug 24;10:961613. doi: 10.3389/fpubh.2022.961613 (PMC9449144; doi:10.3389/fpubh.2022.961613)
Supplement: Supplementary file 1 [file Data_Sheet_1.pdf]

**Supplementary Table 1** Prevalence rates of top five common subtypes of congenital heart defects in Guilin, 2018 – 2020 (per 1000 births)

| Types of BDs | **2018** (N:56006 births) | | **2019** (N:50071 births) | | **2020** (N:41740 births) | | **Total** (N:147817 births) | |
|---|---|---|---|---|---|---|---|---|
| | Prevalence | Rank | Prevalence | Rank | Prevalence | Rank | Prevalence | Rank |
| Ventricular septal defect | 0.45 | 3 | 0.74 | 2 | 0.91 | 3 | 0.68 | 3 |
| Atrial septal defect | 1.68 | 1 | 2.16 | 1 | 2.59 | 1 | 2.10 | 1 |
| Atrioventricular septal defect | 0.50 | 2 | 0.50 | 4 | 0.07 | 5 | 0.38 | 4 |
| Tetralogy of Fallot | 0.05 | 5 | 0.00 | - | 0.10 | 4 | 0.05 | 5 |
| Patent ductus arteriosus | 0.34 | 4 | 0.74 | 3 | 1.37 | 2 | 0.76 | 2 |

**Supplementary Table 2**. Congenital heart defect (CHD) cases counts and proportion at different two diagnosis periods in Guilin, 2018 – 2020

| Diagnosis period | 2018 | 2019 | 2020 | N (%)Total |
|---|---|---|---|---|
| Prenatal diagnosis | 19 | 18 | 14 | 51 (10.85%) |
| Postpartum diagnosis within 7 days | 128 | 154 | 174 | 456 (89.15%) |
| N (%)Total | 147 (28.99%) | 172 (33.93%) | 188 (37.08%) | 507 (100%) |

N: number

**Supplementary Table 3** Neural tube defects (NTD) cases description in Guilin, 2018 – 2020

| Year | Diagnosis (pregnancy outcome) |
|---|---|
| 2018 | Anencephaly with cleft lip and palate (Pregnancy terminations); Anencephaly (Pregnancy terminations); Spina bifida (Pregnancy terminations); Spina bifida associated with congenital atresia of rectum and anus (live birth); Encephalocele with craniotabes (Pregnancy terminations) |
| 2019 | - |
| 2020 | Anencephaly (Pregnancy terminations) |
